# Higher Education

**Published:** 1893-01

**Authors:** 


### EDITORIAL DEPARTMENT.

F. E. DANIEL, M. D., Editor.
A. J. SMITH. M. D., Galveston, Associate Editor.

EDITORIAL STAFF:

PROF. J. E. THOMPSON, M. D., Texas Medical College, Galveston; Surgery.
PROF. WM. KEILLER, M. D., Texas Medical College, Galveston; Obstetrics and Gynecology.
PROF. DAVID CERNA, M. D., Texas Medical College, Galveston; Therapeutics.
PROF. A. J. SMITH, M. D., Texas Medical College, Galveston; Medicine.
DR. ISADORE DYER, Tulane University; Dermatology.
DR. R. H. L. BIBB, Saltillo, Mexico; Foreign Correspondent.
DR. ROBT. MORRIS, Charity Hospital, N. O.; Clinical Reports.

The JOURNAL is the official organ of the Austin District Medical Society, the West Texas District Medical Society and the Galveston County Medical Society.

### HIGHER EDUCATION.

The following bill has been prepared by friends of the Texas University Medical College, and will be introduced in the Legislature now in session at Austin. It is in pursuance of the movement amongst Southern medical colleges, an account of which was given in our December number, looking to raising the standard of medical education throughout the South. It will be remembered, the Association of Southern Medical Colleges agreed to require hereafter a three year's course as a condition to graduation. The Texas Medical College required it from the first, but so long as other Southern schools were making graduation easier and charging lower fees, it was impossible for the Texas Medical College to compete with them for even the Texas patronage. At the present session of the Legislature, an effort will be made also to reduce the fees at the Texas Medical College; this done, say to $50, the fee charged, we believe, by a majority of Southern schools, and all other colleges in the South insisting on the same requirements as these instituted by the Texas Medical College, it is to be hoped another year we will see a better class at Galveston. Texas has equipped and put in operation a magnificent Medical Department of her University, and it is rather humiliating that only twenty-two or three of the Texas students have matriculated there.

This bill will probably be amended in the house. Should it become a law, it will give us better doctors, and, it is hoped, fewer graduates:

